# Anesthetic Management of Left Ventricular Assist Device Exchange in a Patient with Intracranial Hemorrhage

**Published:** 2020-01

**Authors:** Nevriye Salman, Sınan Sabit Kocabeyoğlu, Ülkü Sabuncu, Ümit Kervan, Mustafa Paç, Ayşegül Özgök

**Affiliations:** 1 *Department of* *Anesthesia and Reanimation, SBU Yüksek İhtisas Ankara Research and Training Hospital, Ankara, Turkey.*; 2 *Department of* *Cardiovascular Surgery, SBU Yüksek İhtisas Ankara Research and Training Hospital, Ankara, Turkey.*

**Keywords:** *Heart-assist devices*, *Intracranial hemorrhages*, *Anesthesia*

## Abstract

The gold standard treatment for end-stage heart failure is heart transplantation; however, the rate of transplantation remains inadequate because of the paucity of organ donation. The left ventricular assist device (LVAD) has been used as a bridge therapy before transplantation. The LVAD is being used increasingly because it reduces mortality despite the accompanying morbidities. Therefore, the anesthetic management of LVAD-related morbidities is important and requires experience and knowledge. Herein, we describe a 60-year-old male patient with an LVAD with complaints of right hemiparesis, dysphasia, and facial paralysis. We aim to present the anesthetic management of a patient with intracranial hemorrhage who underwent LVAD exchange due to thrombosis.

## Introduction

Congestive heart failure is a fatal disease with an annual mortality rate of 25%.^1^^, ^^2^ Owing to the large number of patients on waiting lists, the implantation of the left ventricular assist device (LVAD) is successfully performed as a bridge to transplantation or as a destination therapy in patients who cannot undergo transplantation.^2^^-^^4^

Recent years have seen decreases in the mortality rate and increases in quality of life in patients with the LVAD by comparison with medical therapy; nonetheless, stroke, thrombotic events, infection, and bleeding are still significant fatal complications.^3^ After LVAD implantation, neurological complications such as stroke and intracranial hemorrhage are the most common causes of death.^4^ In addition, antiplatelet and anticoagulant therapy that should be used in these patients increase the risk of hemorrhagic complications.^1^

The aim of this report is to describe the anesthetic management during an LVAD pump-exchange procedure of a patient who had pump thrombosis after the termination of anticoagulant therapy due to subarachnoid hemorrhage (SAH). 

## Case Report

A 60-year-old (90 kg, 172 cm) patient was admitted to our emergency department with right hemiparesis, dysphasia, and facial paralysis. The patient had a history of HeartWare (Medtronic Inc.; Framingham, MA, USA) implantation via left anterolateral thoracotomy 1 year previously due to ischemic heart failure.

**Figure 1:**
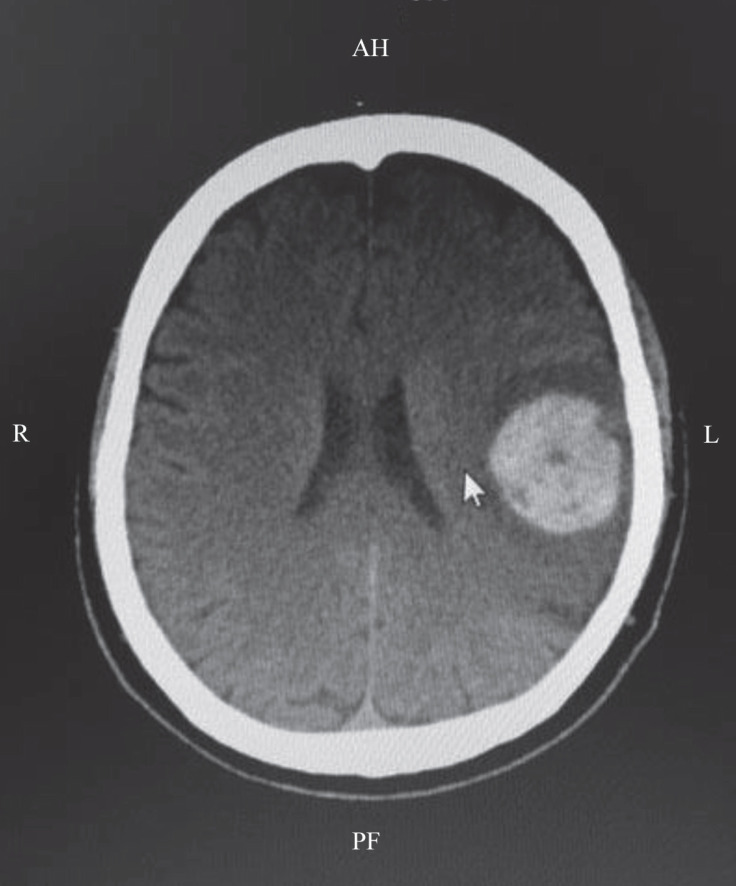
Cranial computerized tomography shows subarachnoid hemorrhage (arrow) in the left central sulcus.

The laboratory tests revealed an international normalized ratio (INR) of 3.23, a prothrombin time of 36%, a hemoglobin level of 12.6 g/dL, and a platelet level of 150×10^3^ /uL. The neurological examination revealed that the patient was confused, with no cooperation and orientation. He had weakness in the right arm and leg (2+/5), dysphasia, loss of blinking control on the right side, decreased tearing, drooping of the mouth to the right side, and a Glasgow Coma Score of 10. Cranial computed tomography (CT) was planned for the patient upon consultation by the neurosurgery department. In the cranial CT, hematoma accompanied by edema in the left parietal lobe and linear densities compatible with SAH in the left superior central sulcus and left ventricular minimal compression were observed, which showed intracranial hemorrhage progression (Figure 1). The LVAD is incompatible with magnetic resonance imaging, and the patient’s unstable hemodynamic state precluded additional diagnostic methods for SAH. 

The neurosurgery department did not plan emergency surgery; anti-edema therapy was administered with dexamethasone and furosemide as suggested. Anticoagulant and antiaggregant therapy was stopped. Subsequently, left ventricular heart failure with pulmonary edema and hemodynamic instability developed in the patient. Pump thrombosis was suspected, and this diagnosis was confirmed by transthoracic echocardiography. Sixteen days after admission to the hospital, emergency HeartWare Ventricular Assist Device (Medtronic Inc.; Framingham, MA, USA) exchange was planned.

In addition to cardiac anesthesia monitorization (electrocardiography, invasive blood pressure, and pulse oximetry), neurological monitoring with near-infrared spectroscopy (NIRS), Pulse index Continuous Cardiac Output (PiCCO) (PULSION Medical Systems, Munich, Germany), and the bispectral index was performed. Due to left heart failure, low-dose inotropic support (0.5 μg/kg/min of dobutamine) was started preoperatively. The patient was taken to the operating room with arterial pressure of 87/42 mmHg, heart rate of 120/min, oxygen saturation (SpO_2_) of 89.2 mmHg, and partial pressure of carbon dioxide (pCO_2_) of 37.5 mmHg. 

Anesthesia induction of the patient was performed with 2 mg/kg of ketamine, 0.2 mg/kg of midazolam, 1 µg/kg of fentanyl, and 0.6 mg/kg of rocuronium. A 40-F left-sided double-lumen endobronchial tube was used for intubation. Thereafter, the bronchial cuff was collapsed, and mechanical ventilation was started with a tidal volume of 4–6 mL/kg, 10–12/min (adjusted according to end-tidal CO_2_ values), and 50% oxygen/air mixture. Anesthesia was maintained with bolus doses of 1 µg/kg of fentanyl, and infusion dosages of 0.05 mg/kg/h of midazolam, and 0.5 mg/kg/min of ketamine to keep the bispectral index values between 40 and 50. At the beginning of the operation, the NIRS values were 52/50 (R/L) and they were maintained at 50/51±10. During the operation, a 0.5-mg adrenaline bolus was administered because of hypotension due to cardiac manipulation and cannulation; the NIRS values dropped to 38/39 for 10 minutes and 1 mg/kg of pentothal was administered for cerebral protection. Afterward, the patient’s blood pressure returned to normal levels, and his NIRS values returned to baseline. Respectively, 0.1 μg/kg/min of noradrenaline and 0.05 μg/kg/min of adrenaline infusions were initiated due to hypotension. Subsequently, the adrenaline infusion was stopped, and a noradrenaline infusion was increased gradually. Because the contractility was adequate, adrenaline was discontinued and noradrenaline was continued for vasoconstriction.

The pulmonary evaluation of the patient revealed bilateral crepitant rales, and the initial arterial blood gas analysis revealed an SpO_2_ level of 89.2 mmHg, a pCO_2_ level of 37.5 mmHg, a hemoglobin level of 10.8 g/dL, and a glucose level of 204 mg/dL. Due to the high blood sugar values, 1 unit/h of insulin infusion was immediately initiated, and the blood glucose concentration was maintained between 100 and 180 mg/dL. Therefore, after heparinization, extracorporeal membrane oxygenation (ECMO) support was commenced for 1-lung ventilation; conventional cardiopulmonary bypass was not preferred on account of the higher dose of heparin. Heparin (5000 U) was applied, and the activated clotting time value was kept as twice the input value. The patient was given 5 packs of erythrocyte suspension, 7 packs of fresh frozen plasma, and 2 packs of pooled platelet suspension. In addition, 250–300 μg/kg of vitamin K and 10 mg/kg of boluses and then 1 mg/kg/h of tranexamic acid infusion were administered to avoid postoperative bleeding. After the LVAD exchange, the patient was weaned from ECMO with 0.3 μg/kg/min of noradrenaline and 3 μg/kg/min of dobutamine infusions. 

**Figure 2:**
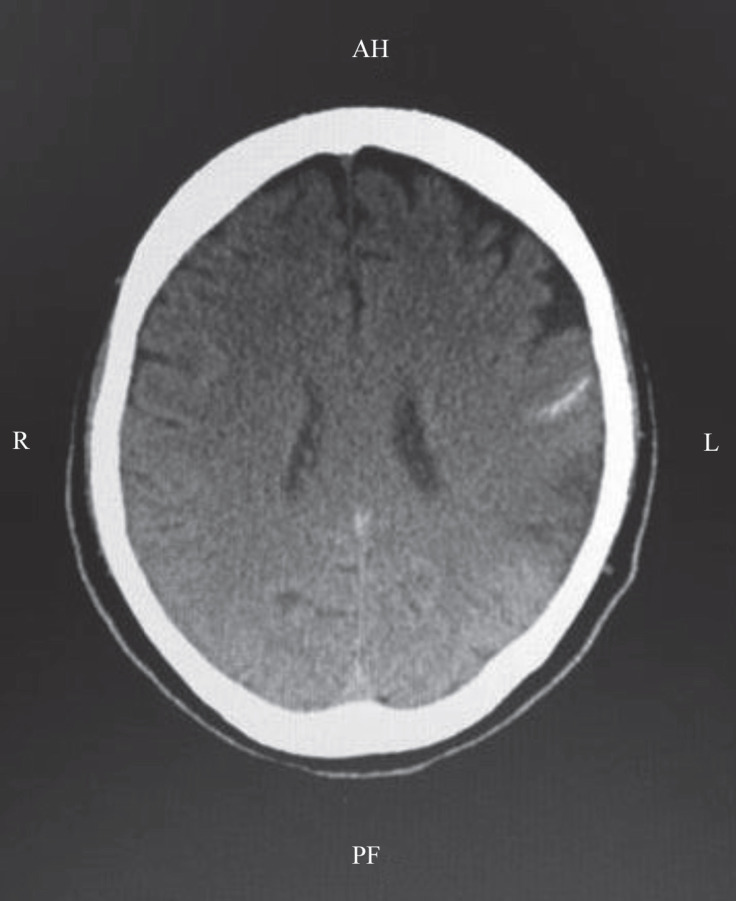
Postoperatively, the intracranial hematoma is almost resolved and the ventricles have returned to normal in computerized tomography.

At the end of the operation, the patient’s double-lumen endobronchial tube was replaced with a single-lumen endobronchial tube and he was transferred to the intensive care unit (ICU). During a postoperative period of 14 days, the hematoma began to resolve and the ventricles were normal (Figure 2). The INR was kept between 2 and 2.5 postoperatively. During the ICU follow-up, the patient was conscious, non-oriented, and cooperative; additionally, he had 4/5 power loss in the right upper extremity and was aphasic. After 1 month of ICU stay, he was transferred to the cardiac surgery ward and was discharged after 3 months, at which time he was fully conscious, oriented, and cooperative and had 4/5 power loss in the right extremity, nasolabial groove deletion, and partial recovery of aphasia. 

## Discussion

The incidence rate of pump thrombosis varies between 0.04% and 0.09%.^2^ Pump thrombosis may present in a wide spectrum, varying from minimal hemolysis/minimal changes in pump parameters to acute hemodynamic collapse, multiorgan failure, massive hemolysis, or critical thromboembolic complications.^2^ In our patient, pump thrombosis caused hemodynamic instability, necessitating inotropic support and an urgent pump exchange.

The treatment of pump thrombosis depends on the localization of the thrombus and the clinical status of the patient.^2^ The treatment options for pump thrombosis are pump exchange and thrombolysis. Due to SAH in our patient, thrombolysis was not an appropriate treatment option and pump exchange was performed. Consequently, resorptions of hemorrhage due to thrombolysis treatment and kidney/multiorgan failure, which can develop secondary to thrombolytic treatment, were also avoided.

A major problem in patients with the LVAD is the difficulty in anesthesia management because of end-stage heart failure; the transition of drugs to the circulation and the distribution of the drugs are decreased when compared with healthy patients owing to the low ejection fraction. Accordingly, more attention should be paid to drug doses and titration. The monitorization of the bispectral index helps to protect cerebral autoregulation by allowing adjustment for adequate blood pressure and anesthetic drug titration.^5^ We, therefore, opted for midazolam and ketamine in our patient for the induction and maintenance of anesthesia to ensure adequate anesthesia depth (bispectral index value = 40–50) and to avoid hemodynamic lability. We also tried to reduce the intracranial pressure-enhancing effect of ketamine by providing low-dose use with anesthesia depth control and the combination with midazolam and fentanyl.

Transesophageal echocardiography and pulmonary artery catheterization are recommended for monitoring hemodynamic data in LVAD intraoperative anesthetic management.^6^ However, given our patient’s abnormal bleeding parameters, we used continuous cardiac output measurement with a PiCCO system. We also utilized the activated clotting time instead of thromboelastography/rotational thromboelastometry, which is recommended for coagulation follow-up,^6^ because it is not available in our hospital.

In patients with intracranial hemorrhage, major precautions that need to be taken include soft intubation, avoidance of hyper/hypotension, mild hyperventilation, head elevation, and avoidance of high concentrations of inhalation agents and nitrous oxide in order not to increase intracranial pressure.^5^ We preferred ECMO support to maintain low intracranial pressure in our patient by preventing hypoventilation and hypercarbia, treated his hypotension with inotropic agents, and performed soft intubation. Additionally, we administered an osmotic diuretic agent (2 mg/kg of 20% mannitol intravenously) to increase the intracranial pressure reduction. 

Although there is no clinically relevant evidence, pharmacological protective agents (thiopental and etomidate) are recommended in ischemia.^5^ Hence, we aimed to use thiopental for cerebral protection, especially in the period when our patient’s NIRS values were reduced. We also administered corticosteroids during the prolonged hypotension periods as is suggested in the literature.^5^ It is known that steroids have many adverse effects including hyperglycemia, but they decrease the proinflammatory response, act as an antioxidant in the ischemic tissue, and allow the regulation of serum sodium and plasma osmolarity.^7^

Excessive blood loss due to high INR values and reoperation can cause hemodynamic instability and ischemia in neurons.^5^ Accordingly, we transfused blood products to provide both normovolemia and normal oxygenation (Hb~10 g/dL) and to prevent adverse effects on cerebral oxygenation due to anemia.^5^ As a result, our patient’s intracranial pressure was reduced and his perfusion pressure was improved.^7^ Following the patient’s hypotensive period, we maintained his mean arterial pressure as high as possible to prevent the adverse effects of ischemia.

Hyperglycemia worsens and prolongs healing in patients with SAH; blood glucose concentrations should, therefore, be treated over 180 mg/dL and maintained between 80 and 200 mg/dL.^5^ In our patient, the blood glucose concentration was maintained between 100 and 180 mg/dL with an insulin infusion.

## Conclusion

Postoperative intracranial hemorrhage is a complication that significantly reduces survival expectancy in patients with the LVAD. Moreover, during pump exchange procedures of patients with intracranial hemorrhage, the method of cerebral protection, the maintenance of hemodynamic stability, and the titration of anesthetic agents may complicate the management of anesthesia and, thus, need experience. In such cases, the treatment and follow-up of multiple parameters, avoidance of hypotension, and the use of neuroprotective methods can prevent possible neurological complications.

## Notes:

***This paper should be cited as: ***Salman N, Kocabeyoğlu SS, Sabuncu Ü, Kervan Ü, Paç M, Özgök A. Anesthetic Management of Left Ventricular Assist Device Exchange in a Patient with Intracranial Hemorrhage. J Teh Univ Heart Ctr 2020;15(1):31-34.
